# Combinatorial regulation of a *Blimp1* (*Prdm1*) enhancer in the mouse retina

**DOI:** 10.1371/journal.pone.0176905

**Published:** 2017-08-22

**Authors:** Taylor S. Mills, Tatiana Eliseeva, Stephanie M. Bersie, Grace Randazzo, Jhenya Nahreini, Ko Uoon Park, Joseph A. Brzezinski

**Affiliations:** Department of Ophthalmology, University of Colorado Denver, Aurora, CO, United States of America; University of Washington, UNITED STATES

## Abstract

The mouse retina comprises seven major cell types that exist in differing proportions. They are generated from multipotent progenitors in a stochastic manner, such that the relative frequency of any given type generated changes over time. The mechanisms determining the proportions of each cell type are only partially understood. Photoreceptors and bipolar interneurons are derived from cells that express Otx2. Within this population, *Blimp1* (*Prdm1*) helps set the balance between photoreceptors and bipolar cells by suppressing bipolar identity in most of the cells. How only a subset of these Otx2+ cells decides to upregulate *Blimp1* and adopt photoreceptor fate is unknown. To understand this, we investigated how *Blimp1* transcription is regulated. We identified several potential *Blimp1* retinal enhancer elements using DNase hypersensitivity sequencing. Only one of the elements recapitulated *Blimp1* spatial and temporal expression in cultured explant assays and within the retinas of transgenic mice. Mutagenesis of this retinal *Blimp1* enhancer element revealed four discrete sequences that were each required for its activity. These included highly conserved *Otx2* and *ROR* (retinoic acid receptor related orphan receptor) binding sites. The other required sequences do not appear to be controlled by Otx2 or ROR factors, increasing the complexity of the *Blimp1* gene regulatory network. Our results show that the intersection of three or more transcription factors is required to correctly regulate the spatial and temporal features of *Blimp1* enhancer expression. This explains how *Blimp1* expression can diverge from Otx2 and set the balance between photoreceptor and bipolar fates.

## Introduction

The seven major neuronal and glial cell types of the retina are derived from a pool of multipotent progenitors in a distinct, but highly overlapping order [1–6]. In the mouse, these cell types can loosely be categorized by whether they permanently exit the cell cycle before or after birth [7–11]. Cone photoreceptors, ganglion, horizontal, and amacrine cells are formed embryonically. The Müller glia, bipolar cells, and rod photoreceptors are mostly formed postnatally. These retinal cell types are also found in drastically different numbers. Their populations range from horizontal interneurons at less than 1% of the retina to rods that make up 78% of the total cells [12]. Cell fate choice in the retina is a stochastic process where the proportion of progenitors adopting any given identity changes over time [1–6, 13]. How this dynamic balance of cell fate outcomes is regulated remains to be fully elucidated.

The transcription factor *Otx2* is expressed broadly in the optic vesicle and early optic cup [14–16]. By the beginning of neurogenesis at embryonic day (E) 11.5, Otx2 is nearly absent from the retina. Corresponding with the onset of photoreceptor formation at E12.5, Otx2 is expressed again, but in retinal progenitors undergoing their terminal division [15–17]. As development proceeds, Otx2 remains expressed in nascent and mature photoreceptors and bipolar cells [15, 16]. Otx2 also permanently marks the retinal pigmented epithelium (RPE) [15], support cells immediately adjacent to the retina that are needed for photoreceptor function. If *Otx2* is genetically removed from the developing retina, extra amacrine cells are formed at the expense of photoreceptors, horizontals, and bipolar cells [18–20]. In addition, these *Otx2* mutants lack expression of the transcription factor *Blimp1* (*Prdm1*) [21]. *Blimp1* is expressed in multiple tissues, but in the retina it is confined to a large subset of Otx2+ cells [21–24]. Its expression is transient, becoming activated at E12.5 and terminating around postnatal day (P) 10 when photoreceptor genesis is complete. Removing *Blimp1* function in the retina causes a one-to-one fate shift of photoreceptors to bipolar interneurons [21–23]. Photoreceptor specification still occurs in these mutants, arguing that *Blimp1* acts negatively to suppress bipolar cell identity. Thus, the balance between photoreceptor and bipolar fates is controlled by Blimp1.

While Blimp1 affects the final fate outcome of Otx2+ cells, it is unclear how these cells decide whether to activate *Blimp1*. Fate mapping experiments show that *Blimp1* expression is not confined to Otx2+ cells that are already committed to photoreceptor identity [23]. This suggests that *Blimp1* is transiently or weakly activated in all Otx2+ retinal cells. *Blimp1* then becomes further upregulated in photoreceptors and downregulated in non-photoreceptor cell types. Achieving this spatial and temporal expression pattern requires the action of Otx2, but also additional transcriptional regulators. The identity of these factors and how they affect *Blimp1* and fate choice is poorly understood.

We reasoned that the choice between photoreceptor and bipolar cell fates could be better understood by uncovering the gene regulatory network responsible for *Blimp1* expression. To identify this network, we used DNase hypersensitivity site (DHS) sequencing of whole retinas to find accessible chromatin regions that could be acting as retina-specific *Blimp1* enhancers [25]. Of the nine DHS peaks we identified and tested for enhancer activity, only one recapitulated *Blimp1* spatial and temporal expression in explant cultures and transgenic mice. Closer examination of the region revealed multiple essential sequences over the span of 139 base pairs (bp). This included a highly conserved *Otx2* binding site, which is consistent with *Blimp1* acting downstream of Otx2 in the retina. We also found a conserved *ROR* (retinoic acid receptor related orphan receptor) binding site and two longer sequence regions (23bp and 40bp) that were required for enhancer activity. These longer regions lacked *ROR* elements and failed to bind Otx2 *in vitro*, arguing that additional transcription factors are required for *Blimp1* enhancer activation. This suggests a more complex combinatorial regulatory model where *Blimp1* enhancer activity is controlled by at least three transcription factors. The intersection of these factors in time and space allow *Blimp1* to be transiently expressed in only a subset of Otx2+ cells. By varying the levels of these upstream activators over time, the choice between bipolar and photoreceptor fates can become dynamic, mirroring what is seen during retinal development.

## Materials and methods

### Enhancer identification and cloning

We searched for DNase hypersensitivity sites (DHS) within approximately 250 kilobases (kb) of the *Blimp1* gene on mouse chromosome 10. ENCODE DHS-seq data [25] was loaded on the UCSC Genome Browser for P0, P7, and P56 whole retina samples. From this, we identified nine candidate DHS sites (A-I) (S1 Fig). DHS-seq data from the ENCODE database for E18.5 brain, P56 brain, cerebellum and activated T-regulatory cells was used for cross-comparison.

To evaluate genomic sequences for enhancer activity, we inserted each of them (S1 Table) upstream of a TATA minimal promoter driving nuclear localized GFP (nGFP) expression using InFusion (Clontech, Mountain View, CA, USA) cloning according to the manufacturer’s instructions [25]. The resulting plasmids were verified by Sanger sequencing. A construct that expressed nuclear Cherry ubiquitously through an Ef1α regulatory element was used as an electroporation control [25]. The TATA-nGFP plasmid lacking any enhancer sequences was used as a control for background vector expression. Numerous enhancer F sub-elements were similarly cloned into the TATA-nGFP plasmid, as above (S1 Table). For site directed mutagenesis, InFusion was used according to the manufacturer’s instructions, replacing the sequence with a string of adenines (A’s) (S1 Table). Since the *Blimp1* promoter lacks a TATA box, we also modified the TATA-nGFP vector to contain the 50 bases up- and downstream of the *Blimp1* transcription start site (±50-nGFP) in lieu of the TATA box. Just the nine DHS sequences (A-I) were cloned into ±50-nGFP construct, as above.

### Explant cultures, electroporations, and quantification

Potential enhancer elements were screened in newborn explants cultured for 1 day *in vitro* (DIV) with some modifications from prior work [25]. Briefly, newborn *C57BL/6* or *CD1* mouse retinas were dissected into HBSS+ (HBSS with Ca2+ and Mg2+, 6 mg/ml glucose, and 0.05M HEPES) on ice. The retinas were transferred into PBS without Ca2+ or Mg2+ for electroporation. A 1:1 ratio of enhancer-nGFP and Cherry control plasmids (1.5 μg/μL each) in 30% glycerol with methyl green was made. The retinas were placed photoreceptor side-up, and 2 μL of the DNA mixture was pipetted on top of them. The explants were individually electroporated with a BioRad Gene Pulser Xcell (Biorad, Hercules, CA, USA) set to deliver five square wave pulses (50V for 50 ms with 250 ms gap intervals). Retinas were transferred to 1 mL of culture media (Neurobasal media, 1X N2 supplement, 1X L-glutamine, 1X penicillin/streptomycin, and 1% FBS) (Gibco/ThermoFisher Scientific, Waltham, MA, USA) in a 12 well plate and cultured for 24 hours at 37°C with 5% CO_2_ and constant gentle mixing (Nutator, 12 RPM).

We quantified the number of Cherry+ cells and GFP+ cells that co-expressed Blimp1 in 1 DIV explant cultures. We counted a minimum of six images from three retinal explants for each condition. This represented a total of 429 images and thousands of GFP+ and Cherry+ cells. From this, we calculated the average percentage of cells that co-expressed Blimp1 and the standard deviation (S.D.). The average percentage and S.D. for Cherry+ cells was calculated from 78 images from TATA-nGFP, F, and F derivative electroporations. We used unpaired t-tests to compare the percentage of GFP+ cells that co-expressed Blimp1 to either Cherry+/Blimp1+ or TATA-nGFP+/Blimp1+ conditions. A P <0.05 was considered significant. To compare GFP+/Blimp+ double labeled percentages between constructs, we used one-way ANOVA and considered a P <0.05 significant.

For long-term cultures, explants were electroporated as above and then flattened ganglion-side up onto 0.4 μm Milicell CM cell culture inserts (Milipore, Billerica, MA, USA) and cultured at the air-media interface. Half of the media was changed every other day and explants were collected at 10 DIV.

### Immunohistochemistry and imaging

Retinal explant and transgenic mouse (below) tissue was fixed in 2% paraformaldehyde for 30 to 120 minutes at room temperature, cryopreserved through 30% sucrose, and frozen in OCT (Sakura Finetech, Torrance, CA, USA). Cryosections were cut at 10 μm and immunostained as previously described [22, 23]. The following primary antibodies were used: rat anti-Blimp1 (1:100) (sc47732, Santa Cruz, Dallas, TX, USA); chicken anti-GFP (1:1000) (ab13970, Abcam, Cambridge, MA, USA); goat anti-Otx2 (2.5μg/mL) (BAF1979, R&D Systems, San Jose, CA, USA); rabbit anti-Pax6 (1:500) (901301, BioLegend, San Diego, CA, USA); guinea pig anti-Ptf1a (1:500) (a gift from Jane Johnson, UTSW); rabbit anti-red fluorescent protein (1:500) (ab34771, Abcam); goat anti-Sox2 (1:100) (sc17320, Santa Cruz); and sheep anti-Vsx2 (1:200) (X1179P, Exalpha, Shirley, MA, USA). Three to five z-stack images were collected using an Olympus FV1000 (Waltham, MA, USA) or Nikon C2 (Melville, NY, USA) laser scanning confocal microscope. Maximum intensity projection images were generated with ImageJ [26] and minimally processed with Adobe Photoshop (San Diego, CA, USA).

### Transgenic mouse construction and analysis

All mice were used in accordance with procedures approved by the University of Colorado Denver IACUC. The F3-TATA-nGFP plasmid lacking backbone vector sequences was used to create transgenic mice on the *C57BL/6* background through standard pronuclear injection with the assistance of the University of Colorado Denver Bioengineering Core. Offspring were genotyped by PCR using Pk3 F and MinGFP-R primers (S1 Table) at 59°C to yield a 646bp product. The resulting four positive founders were bred to *C57BL/6* mice. Tissues from embryos and postnatal mice were used for immunohistochemical analysis (see above). Only one of the four founders showed GFP expression and was used for all subsequent analysis. For each time-point, three *F3-nGFP* transgenic mice were examined. To calculate double and triple labeled populations at E15.5 and P0, the percentage of GFP+ cells that co-expressed a given marker was calculated from a minimum of three mice and eight images. This represented a total of 35 images and 4,135 GFP+ cells counted. For native GFP fluorescence imaging, exposure settings were kept the same for all images.

### Enhancer binding assays and Western blots

Nuclear proteins were purified from newborn wild-type CD1 retinas using a NE-PER nuclear lysis kit (#78833, ThermoFisher) according to the manufacturer’s instructions. Sense oligonucleotides (oligos) to the F3.1d enhancer sequence, sub-elements, and mutants were synthesized and biotinylated on the 5’ end (IDT, San Diego, CA, USA) (S1 Table). Unlabeled antisense oligos were mixed one-to-one with the labeled oligos, heated to 95°C, and allowed to slowly cool to room temperature to make double stranded DNA. We mixed 60 pMol of double stranded oligos with 0.3 mg of streptavidin-coated dynabeads (#11206D, M-280, Invitrogen/ThermoFisher) and rotated them for one hour. The beads were washed according to the manufacturer’s instructions for nucleic acid applications. Oligo-bound (and no-oligo control) beads were incubated with 40 μL of P0 retinal nuclear lysate (above) (~1 μg/μL) diluted to 200 μL in RIPA buffer (140 mM NaCl, 10 mM Tris, 1 mM EDTA, 0.5 mM EGTA, 1% Triton X100, 0.1% deoxycholate, 0.1% SDS) overnight at 4°C with constant rotation. Reactions were washed three times using RIPA buffer and the proteins boiled from the beads under reducing conditions and used for SDS-PAGE. For visualization, one gel with 90% of the boiled protein was stained with Colloidal Coomassie stain (#161–0803, Biorad) according the manufacturer’s instructions. For Western blotting, 10% of the boiled protein sample was subjected to SDS-PAGE and the protein transferred to PVDF membranes. The membrane was blocked for 1 hour in 5% BSA/TBST, and incubated with goat anti-Otx2 (0.5 μg/mL, BAF1979, R&D Systems) antibody in 0.5% BSA/TBST overnight at 4°C. The membrane was incubated with anti-goat-HRP secondaries (1:10,000, Santa Cruz) and developed with Clarity Western ECL substrate (Biorad). Coomassie and Western images were captured with a Biorad ChemiDoc XRS+ system.

### Chromatin immunoprecipitation (ChIP)

Otx2 ChIP was performed on newborn retinal tissue from *C57BL/6* mice as described previously [23]. Quantitative PCR was run with SsoFast EvaGreen Supermix (Biorad) according to manufacturer’s instructions using a Biorad CFX Connect quantitative PCR machine. Primers to *Id3* (negative control) and *Rbp3* (Irbp) (positive control) were used previously [23] and are listed in the supporting information (S1 Table). Primers for the *Blimp1* enhancer started 59bp upstream of the Otx2 “A” site (Otx2 A F) and ended 61bp downstream (Otx2 A R) (S1 Table). Percent of input was calculated as 2^((Ct-input)-(Ct-IP))^ x 100%, where Ct is the threshold cycle. ChIP reactions were conducted three independent times and statistical differences were calculated using unpaired t-tests with P <0.05 considered significant. We were unable to achieve reproducible ChIP results with antibodies to RORβ (sc-21354, Santa Cruz) (14054b, Abgent, San Diego, CA, USA).

### Transcription factor binding predictions

We used the JASPAR (http://jaspar.genereg.net/) [27, 28] database to predict transcription factor binding to the *Blimp1* enhancer region. The 139bp F3.1d sequence was scanned in the core vertebrata model with a relative profile score threshold of 90%. To find additional transcription factor candidates, the database was also searched with a score threshold of 80%. The output is listed in the supporting information (S2 Table). We compared the predictions made by JASPAR to our previously generated RNA-seq data for whole P2 retina [29]. Transcription factors that were not expressed in the P2 retina, such as *Hox* and *Gata* genes, were eliminated from consideration. For simplicity, we grouped transcription factors where one or more family members are predicted by JASPAR and expressed in the retina, such as *Sox* and *Lhx* genes.

## Results

### Identification of potential *Blimp1* enhancer elements

The transcription factor *Blimp1* is required for normal photoreceptor development, but does not specify rod or cone fate [21–23]. *Blimp1* is genetically downstream of *Otx2*, a transcription factor required for the genesis of multiple cell types in the eye [18–21]. However, Blimp1 is only transiently expressed by a subset of Otx2+ cells during retinal development. This suggests that transcriptional regulators besides Otx2 are required for proper *Blimp1* expression and control of photoreceptor fate. To identify these regulators, we searched for non-coding DNA elements (enhancers) that could recapitulate *Blimp1* expression in the retina.

**Fig 1:**
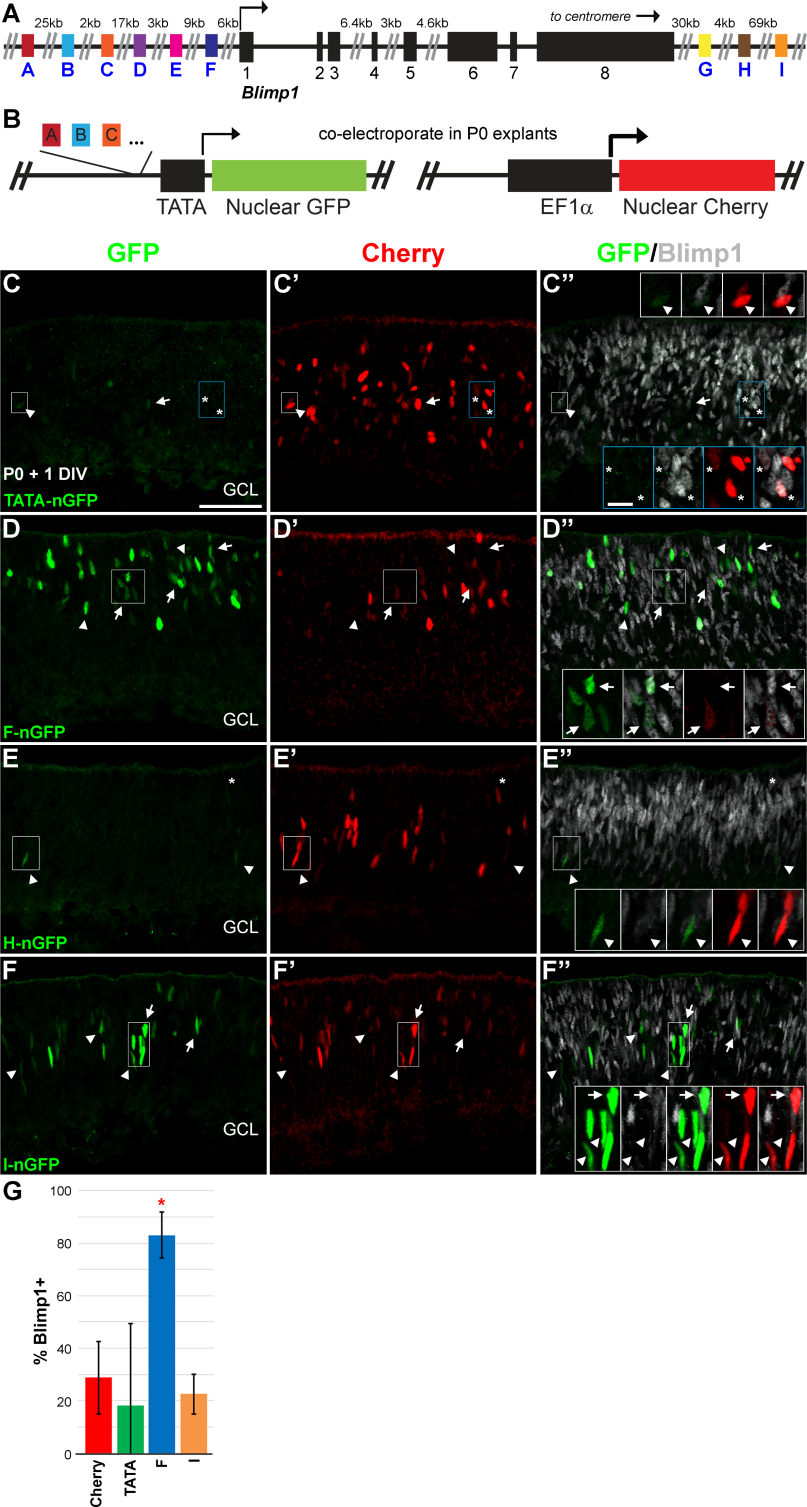
Screen for *Blimp1* retina-specific enhancers. **(A)** Schematic of the *Blimp1* genomic locus on mouse chromosome 10 showing nine DNase hypersensitive sites (DHS) near the gene. **(B)** Schematic of the retinal explant enhancer assay. The nine DHS sequences (A-I) are inserted upstream of a TATA box minimal promoter driving nuclear-localized GFP (nGFP). A separate plasmid contains the ubiquitous EF1α regulatory element driving nuclear-localized Cherry expression. Constructs are co-electroporated into P0 retinal explants and collected for histological analysis after one day *in vitro* (DIV). **(C-C”)** Co-electroporation of TATA-box only (no enhancer) and cherry plasmids. GFP+ nuclei (green) are rarely observed and express GFP at low levels. In contrast, Cherry+ nuclei (red) are widely distributed. Most GFP+ cells do not co-express Blimp1 (grey, arrowheads, insets), but about 25% are co-labeled (arrows). A similar fraction of Cherry+ cells co-express Blimp1 (asterisks, insets). This represents the probability that electroporated plasmids will end up in Blimp1+ cells by chance after 1 DIV. **(D-D”)** Element F drives strong GFP expression in explants and most of these cells co-express Blimp1 (arrows, insets). Arrowheads mark GFP+ cells that do not co-express Blimp1. **(E-E”)** Element H drives little GFP expression and the few positive cells do not co-express Blimp1 (arrowheads, insets). Blimp1+ cells are electroporated, as Cherry+ cells co-express Blimp1 (asterisks). **(F-F”)** Element I showed robust GFP expression with nuclei localized to the middle of the retina. Some GFP+ cells co-express Blimp1 (arrows, insets), but about 80% are Blimp1 negative (arrowheads, insets). GCL, ganglion cell layer. Scale bar is 50 μm for panels and 10 μm for insets. **(G)** Plot of the average percentage of GFP+ or Cherry+ cells that co-express Blimp1 for the active elements. Error bars represent the standard deviation (S.D.). Only element F shows a significant enrichment of Blimp1 co-expression compared to Cherry and TATA box GFP controls (*, unpaired t-test, P <0.001). The high standard deviation in the TATA only condition is due to the paucity of GFP+ cells.

Potential enhancers flanking the *Blimp1* locus on mouse chromosome 10 were identified using previously published DNase hypersensitivity site (DHS) sequence mapping from whole retinal tissue [25]. We focused our search within roughly 250kb of the *Blimp1* gene as BAC transgenic mice closely recapitulate *Blimp1* retinal expression [23]. We examined DHS data from three whole retinal tissue sets; P0, P7, and P56. From this, we identified nine regions (A through I) as candidate enhancers (S1 Fig and Fig 1A). Since *Blimp1* is expressed from embryonic day (E) 12.5 to about P10, we were particularly interested in the DHS peaks that became attenuated in the P56 dataset. Of the nine sites, six (B, C, D, E, G, and H) clearly showed this pattern (S1 Fig). To determine if the DHS sites were retina-specific, we compared DHS data from retina to ENCODE (encyclopedia of DNA elements) data from samples where *Blimp1* is (T-regulatory cells) [30] and is not (E18.5 brain, adult brain, adult cerebellum) expressed (S1 Fig). The three brain samples showed almost no DHS site overlap with retina, as expected for tissues that do not express *Blimp1*. In Blimp1+ activated T cells, DHS overlap was seen only with sites A and G (S1 Fig). Taken together, these data suggest that DHS sites B-F and H-I are retina-specific.

To test whether any of these potential enhancers recapitulated *Blimp1* expression in the retina, we cloned all the DHS peak sequences (S1 Table) into a previously generated [25] minimal promoter (TATA box) plasmid that drives nuclear localized GFP expression (Fig 1B). These constructs were electroporated into P0 retinal explants, a stage when *Blimp1* is robustly expressed. We co-electroporated a plasmid encoding nuclear localized Cherry under the control of a ubiquitously expressed regulatory element to mark the region that was electroporated (Fig 1B). Explants were collected after one day in culture and immunostained for Blimp1, GFP, and Cherry (Fig 1C–1G). Overlap with Blimp1 was readily quantifiable as each marker was nuclear localized. We quantified the percentage of Cherry+ nuclei that co-expressed Blimp1 (Fig 1G). This value (28.8% ± 13.8% S.D.) represented the fraction of electroporated cells that co-express Blimp1 by chance after 24 hours. We then compared the percentage of GFP cells that co-expressed Blimp1 for each enhancer element to the Cherry values. First, we examined retinas electroporated with the minimal promoter construct lacking an enhancer element. This TATA-nGFP construct only weakly drove GFP expression and few positive nuclei were seen in the retina (Fig 1C–1C”). These GFP+ cells were not enriched for Blimp1 co-expression versus Cherry+ cells (Fig 1G). The standard deviation was large because of the paucity of GFP+ cells in any given image. These results show that the TATA minimal promoter can only weakly drive GFP expression and is not preferentially activated in Blimp1+ cells. Next, we tested the nine candidate enhancer elements in our explant system (Fig 1D–1F” and data not shown). Most of the enhancer elements either lacked GFP expression or showed sparse weakly positive cells similar to the TATA vector control (Fig 1E–1E” and data not shown). Only two elements showed robust GFP expression in the retina. Enhancer F drove GFP expression in the outer aspect of the retina, where developing photoreceptors reside (Fig 1D–1D”). Enhancer F-GFP+ cells co-expressed Blimp1 highly (83.1% ± 8.7% S.D.), representing a significant enrichment over Cherry electroporated cells (unpaired t-test, P < 0.001) (Fig 1G). In contrast, enhancer I-GFP+ cells were localized to the middle of the retina and only modestly co-expressed Blimp1 (22.5% ± 7.6% S.D.) (Fig 1F–1F”). This was not significantly different than the value for Cherry (Fig 1G). Element F was the only DHS site that showed evidence of being a *Blimp1* enhancer in our assay, which was somewhat surprising as it still had signal in the P56 dataset (S1 Fig).

We reasoned that since *Blimp1* does not contain a TATA-box promoter, the sequences flanking the transcription start site may be needed for the other candidate enhancers to activate transcription in our assay. To test this, we redesigned our minimal promoter GFP construct to contain the 50 bases both up and downstream of the *Blimp1* transcription start site and reexamined all nine elements in explants. We observed the same expression patterns seen with the TATA box based vectors (data not shown). These data suggest that the elements do not require a specific sequence in the *Blimp1* promoter to activate transcription.

### A conserved part of element F recapitulates *Blimp1* expression *in vivo*

**Fig 2:**
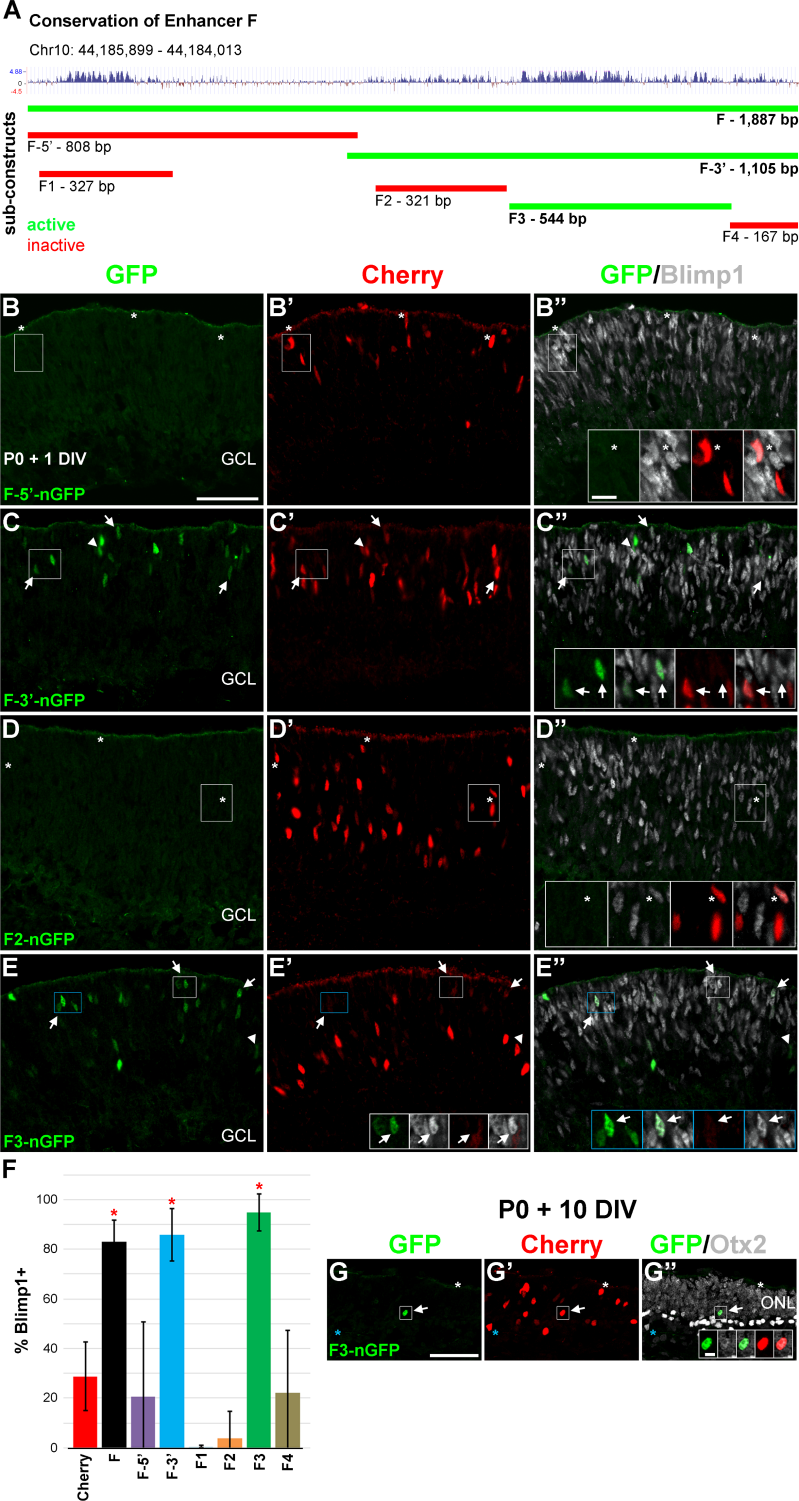
A portion of the F sequence is sufficient to drive expression in Blimp1+ cells. **(A)** Schematic of the 1,887bp F element showing vertebrate conservation. Upward peaks indicate higher conservation. Genomic coordinates are from the mouse mm9 assembly. Six sub-elements are shown to scale and color-coded based on their activity (green, active) (red, inactive) in explant assays. **(B-B”)** Explants co-electroporated with F-5’-nGFP and Cherry. GFP+ cells (green) are rare, but about a quarter of the Cherry+ cells (red) co-express Blimp1 (grey, asterisks, insets). **(C-C”)** The F-3’-nGFP construct is active and most of the GFP+ cells co-express Blimp1 (arrows, insets). Arrowheads mark GFP+ cells that do not co-express Blimp1. **(D-D”)** The F2 element has no activity. Asterisks (insets) show Cherry electroporated cells that co-express Blimp1. **(E-E”)** The F3 construct is highly active and about 90% of GFP+ cells co-express Blimp1 (arrows, insets). Arrowheads mark the few GFP+/Blimp1 negative cells. Scale bars are 50 μm for panels and 10 μm for insets. **(F)** Plot of the average percentage of Cherry+ or GFP+ cells that co-express Blimp1. Error bars represent the S.D. The F-3’ and F3 constructs show significantly more Blimp1 co-expression compared to cherry controls (*, unpaired t-test, P < 0.001). The F3 element also shows modestly, but significantly more Blimp1 overlap than the F parent element (unpaired t-test, P < 0.03). **(G-G”)** The F3 construct examined after 10 DIV, corresponding to when Blimp1 is no longer expressed in the retina. GFP+ cells (arrows, insets) are rarely seen, but are located in the outer nuclear layer (ONL) where Otx2+ (light grey) photoreceptors reside. The white asterisks mark a Cherry+/Otx2+ photoreceptor and the blue asterisks mark a Cherry+/Otx2+ bipolar cell (intense grey). Scale bars are 50 μm for panels and 5 μm for insets.

We reasoned that only a small fraction of the 1,887bp DHS F region was needed to recapitulate *Blimp1* expression. We examined evolutionary sequence conservation and identified two relevant areas (Fig 2A). This included a short stretch of highly conserved sequence in the 5’ region of the element and a much broader area covering the 3’ half. To test what sequences were required, we divided element F into a series of six derivatives and cloned them into the TATA box GFP construct described above (S1 Table and Fig 2A). We first electroporated constructs that divided enhancer F in half. We observed that the 5’ half (F-5’) was unable to drive GFP expression in the retina (Fig 2B–2B”). A sub-fragment of this region (F1) was similarly unable to drive GFP expression. In contrast, the 3’ half (F-3’) was expressed and overlapped highly with Blimp1 (85.9% ± 10.6% S.D.) (Fig 2C–2C” and 2F). This was significantly enriched versus Cherry (unpaired t-test, P < 0.001) and not appreciably different than the value for the entire F element (Fig 2F). We next divided the 3’ sequence into three fragments (F2, F3, and F4) based on conservation (Fig 2A). Elements F2 (Fig 2D–2D”) and F4 (data not shown) were poorly expressed and did not show Blimp1 enrichment (Fig 2F). Element F3, which included the most conserved sequence in the 3’ region, was robustly expressed (Fig 2E–EE”). Nearly all of the GFP+ cells overlapped with Blimp1 (94.8% ± 7.4% S.D.), which was significantly enriched versus Cherry control (unpaired t-test, P < 0.001) and similar to the parent F-3’ element (Fig 2F). This 544bp enhancer F3 region accurately recapitulated the Blimp1 spatial pattern at P0. Since *Blimp1* expression is absent by P10 [21, 22], we reasoned that GFP expression would be transient. To test this, we electroporated retinas and cultured them as flatmounts for 10 DIV. As expected, F3-GFP+ cells were nearly absent from these long-term cultures, but the occasional positive cell was observed (Fig 2G–2G”). Thus, the 544bp F3 enhancer element recapitulates *Blimp1* spatial and temporal expression in explants.

**Fig 3:**
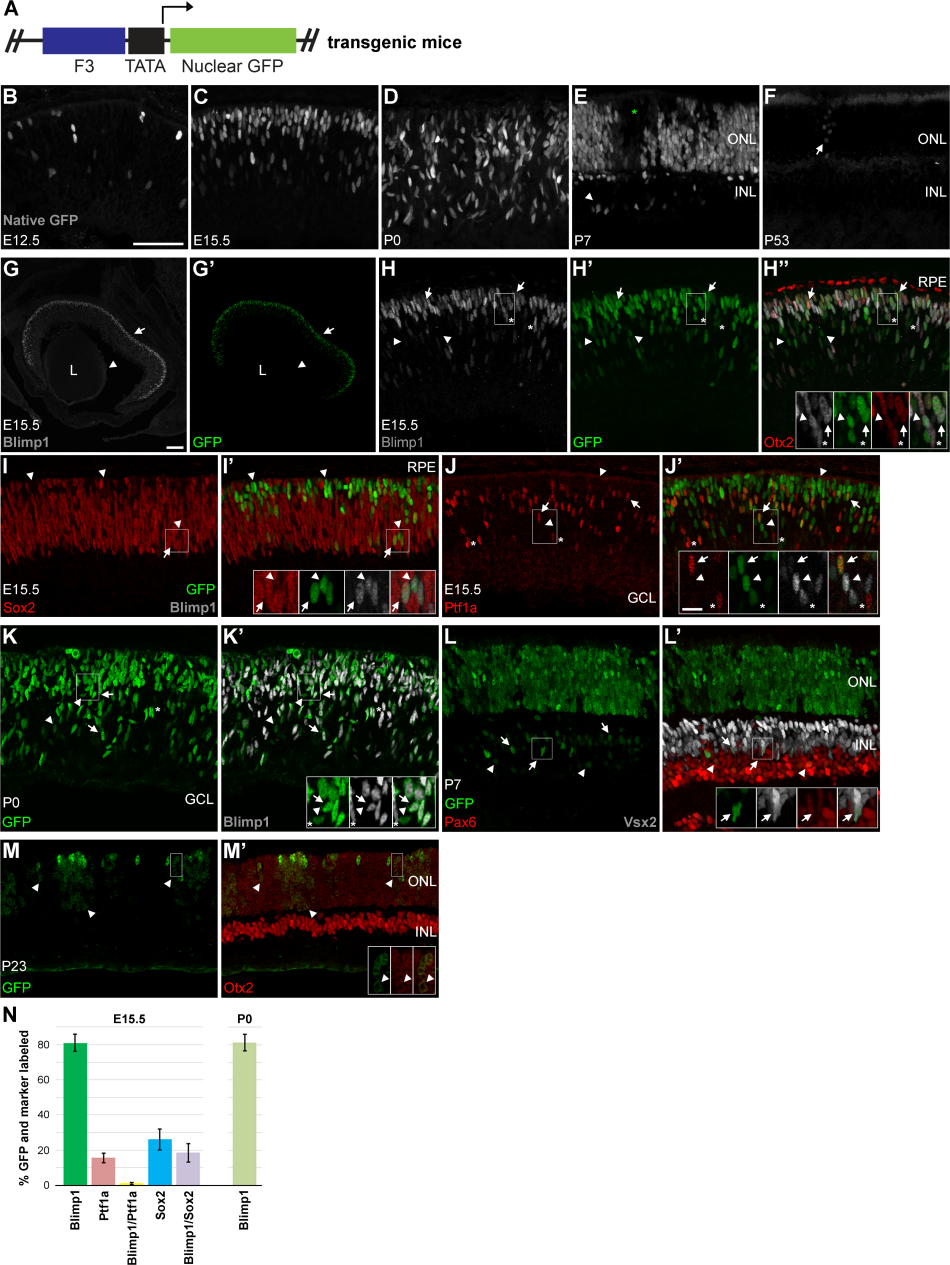
F3-nGFP transgenic mice recapitulate Blimp1 retinal expression *in vivo*. **(A)** Schematic of the construct used to generate transgenic mice. **(B-F)** Native GFP fluorescence (grey) at multiple developmental stages. GFP is first seen in the retina at E12.5 (B), similar to Blimp1 expression. The number of cells expressing GFP at E15.5 (C) and P0 (D) progressively increases. GFP is still expressed at P7 (E), when Blimp1 protein is becoming downregulated. Signal is mostly in the ONL, but some cells in the inner nuclear layer (INL) are evident (arrowheads). Some transgene mosaicism is seen (green asterisk). At P53, weakly GFP+ cells are occasionally seen in the ONL (arrow). **(G-J’)** E15.5 F3-nGFP transgenic mice stained for cell type-specific markers (red, grey). **(G-G’)** Low-power view of the eye. The GFP signal (green) closely matches the Blimp1 immunostaining pattern (grey, arrows). No GFP is seen in the lens (L) or surrounding tissues. Arrowheads mark Blimp1+ vascular endothelial cells in the hyaloid vasculature. These do not co-express GFP, demonstrating retinal specificity of the transgene. **(H-H”)** High-power view of sections co-stained for Blimp1 (grey) and Otx2 (red). Most GFP+ cells co-express Blimp1 and Otx2 (arrows, insets). About 20% of GFP+ cells do not co-express Blimp1 (arrowheads, insets). A small number of Blimp1+/Otx2+ cells do not express GFP (asterisks, insets). The Otx2+ retinal pigmented epithelium (RPE) does not express GFP or Blimp1. **(I-I’)** Sections co-stained for Blimp1 (grey) and the progenitor marker Sox2 (red). Most GFP+ cells do not express Sox2 (arrowheads, insets). About 25% of GFP+ cells co-express Sox2, but most of these also co-express Blimp1 (arrows, insets). Thus, few progenitors (Sox2+/Blimp1-) express GFP. **(J-J’)** Roughly one in six GFP+ cells co-express the amacrine and horizontal cell precursor marker Ptf1a (red, arrows, insets). Unlike Sox2, few of these GFP+/Ptf1a+ cells co-express Blimp1 (grey). Arrowheads (insets) mark GFP+/Blimp1+ cells that do not co-express Ptf1a while asterisks (insets) mark Ptf1a+/GFP- cells. **(K-K’)** At P0, the majority of GFP+ cells co-express Blimp1 (arrows, insets). About 20% of GFP+ cells lack Blimp1 co-expression (arrowheads). Asterisks mark the uncommon Blimp1+ cells that do not co-express GFP. No GFP is seen in the RPE. **(L-L’)** GFP expression is widespread at P7 and predominately labels photoreceptors in the ONL. Weaker INL staining overlaps with either Pax6+ amacrines (red, arrowheads, insets) or Vsx2+ bipolars (grey, arrows, insets). No staining of the RPE or vascular endothelial cells is seen. **(M-M’)** Blimp1 is no longer expressed at P23, but GFP+ cells are still detectable by immunostaining. GFP expression levels are modest and the signal overlaps with Otx2 (red) in the ONL (arrowheads, insets). Mosaic GFP expression is more common and pronounced in older retinas. Scale bars are 50 μm for panels B-F, H-M’, 10 μm for insets, and 100 μm for G, G’. **(N)** Plot showing the average percentage of GFP+ cells that co-express cell type-specific markers at E15.5 and P0. Error bars represent the S.D.

To test whether the F3 element acts as a *Blimp1* enhancer *in vivo*, we constructed transgenic mice (Fig 3A). We obtained four founder lines, but we confined our detailed analysis to one of them as the remaining lines lacked GFP expression. We examined transgenic mice at multiple developmental stages for native GFP fluorescence. At E11.5, before *Blimp1* expression onset, we did not observe any GFP in the eye (data not shown). Starting at E12.5, we observed a small number of GFP+ cells in the outer aspect of the central retina (Fig 3B), similar to Blimp1 staining at this age [21, 22]. As development proceeded, the number of GFP+ cells increased (Fig 3C and 3D). There was no labeling of the RPE or other ocular structures. We did not see GFP fluorescence in embryonic blood vessels, where Blimp1 was previously observed [22]. With the exception of the developing pineal gland, we did not observe GFP fluorescence outside the eye at embryonic stages (data not shown). At P7, when Blimp1 protein is becoming downregulated, we observed GFP fluorescence in the photoreceptor area of the retina (Fig 3E). A few GFP+ cells were also seen in the inner nuclear layer (INL). By the adult stage, little native GFP fluorescence was detected (Fig 3F). When observed, this fluorescence was confined to the outer nuclear layer (ONL) where photoreceptor nuclei reside. These data suggest that the F3 element closely recapitulates spatial and temporal aspects of retinal *Blimp1* expression.

To better characterize the F3 transgenic mice, we immunostained sections at various ages with Blimp1 and other cell type-specific markers. At E15.5, GFP expression was limited to the retina and overlapped extensively with Blimp1 (81.0% ± 4.9% S.D.) (Fig 3G–3H” and 3N). No GFP was seen in Blimp1+ fetal vasculature (Fig 3G and 3G’). As Blimp1 is made in Otx2+ cells, we observed a high degree of overlap between GFP and Otx2 (Fig 3H–3H”). Otx2+ RPE cells do not express Blimp1 and were GFP negative (Fig 3H–3H”). Since only about 80% of the GFP+ cells co-expressed Blimp1 at this age, we examined other cell type-specific markers. We observed moderate GFP overlap with Sox2 (26.2% ± 6.0% S.D.) (Fig 3I, 3I’ and 3N), which is primarily a marker of progenitors at this age [31]. However, most of these double positive cells also co-expressed Blimp1 (18.5% ± 5.3% S.D.) (Fig 3I, 3I’ and 3N). This pattern is consistent with prior data showing that Blimp1 is activated during the last cell cycle [21, 22] and argues that there is little non-specific progenitor GFP expression. We next looked at Ptf1a, a marker of horizontal and amacrine cell precursors [32]. Previous lineage tracing experiments argued that *Blimp1* is transiently made in these precursors [23], which could account for the GFP+ cells that do not express Blimp1. We observed modest co-labeling of GFP and Ptf1a (15.6% ± 2.7% S.D.) at E15.5 (Fig 3J, 3J’ and 3N). Unlike the Sox2 staining, we saw very few cells that co-expressed GFP, Ptf1a, and Blimp1 (1.1% ± 0.5% S.D.) (Fig 3J, 3J’ and 3N). Thus, most of the GFP+ cells that lack Blimp1 appear to have committed to the Ptf1a+ amacrine/horizontal cell lineage, consistent with *Blimp1* lineage tracing experiments. This is likely detectable due to the long half-life of GFP compared to Blimp1 protein. At P0, GFP signal was confined to the neural retina and most of the GFP+ cells co-expressed Blimp1 (81.2% ± 4.7% S.D.) (Fig 3K, 3K’ and 3N). GFP was detectable at P7 when Blimp1 protein levels are low, which was again likely because of GFP stability (Fig 3L and 3L). The majority of the GFP signal was in the ONL, but some cells in the INL were labeled. We co-labeled sections with antibodies to Pax6 and Vsx2 to label amacrines and bipolar cells, respectively [33–36]. We observed GFP+ cells that overlapped with Pax6 or Vsx2 (Fig 3L and 3L’). This is consistent with our data showing that some amacrine precursor cells transiently make GFP and our prior lineage tracing experiments showing that both amacrines and bipolar cells transiently express *Blimp1* [23]. Lastly, we immunostained P23 transgenic mice with GFP and Otx2 to mark photoreceptors and bipolar cells (Fig 3M and 3M’). GFP expression levels were modest compared to younger time-points, but some cells were always seen at this age. GFP+ cells co-expressed Otx2 and were nearly always in the ONL, indicating that GFP is marking photoreceptors. In summary, the F3-GFP transgenic mouse closely recapitulated the spatial and temporal aspects of *Blimp1* retinal expression *in vivo*. This suggests that the F3 element is a retina-specific *Blimp1* enhancer.

### Multiple sites within the *Blimp1* enhancer element are required for its activity

**Fig 4:**
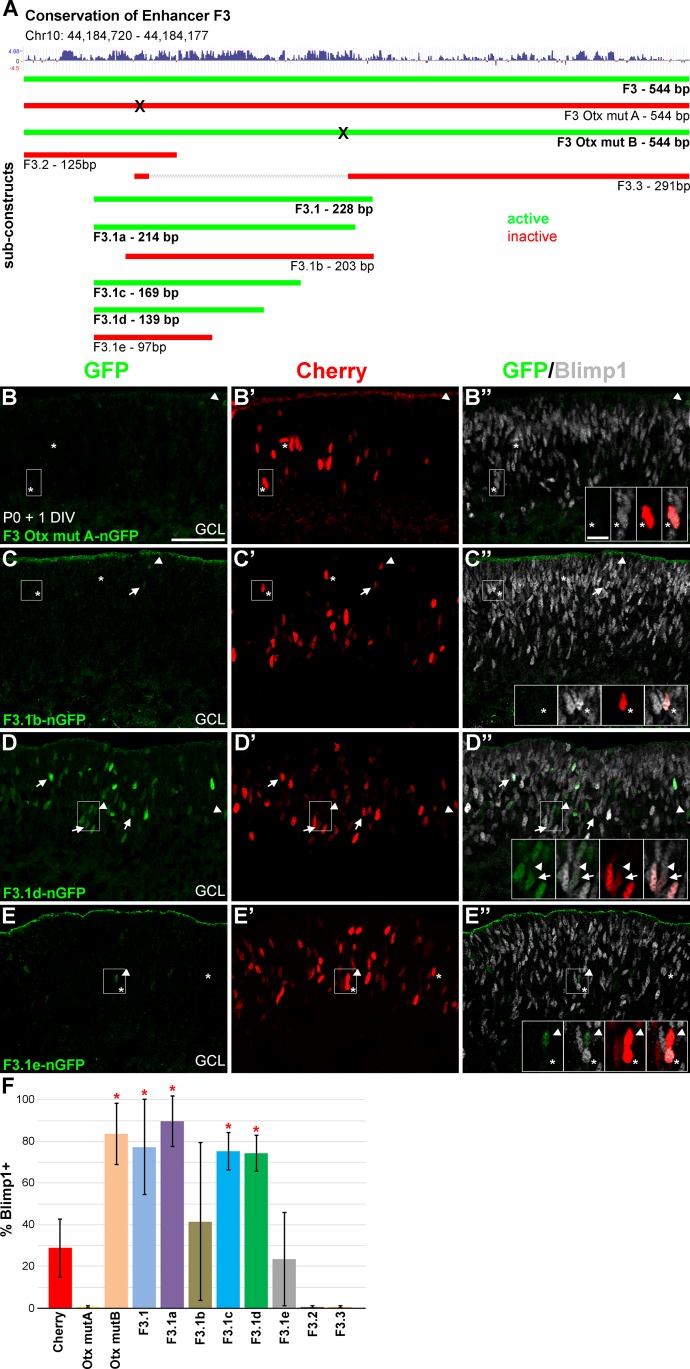
A 139bp region of element F3 recapitulates *Blimp1* expression. **(A)** Schematic of the 544bp F3 region showing vertebrate conservation. Upward peaks indicate higher conservation. Genomic coordinates are from the mouse mm9 assembly. Ten sub-elements are shown to scale and color-coded based on their expression in explants cultured for 1DIV (green, active) (red, inactive). Mutagenesis of *Otx2* sites to 6 A’s is marked with an “X”. **(B-B”)** Mutagenesis of the 5’ *Otx2* “A” site blocks F3 activity and the rarely seen GFP+ cells (green, arrowheads) do not co-express Blimp1 (grey). Asterisks mark Cherry+ cells (red) that co-express Blimp1 (insets). **(C-C”)** The F3.1b sub-element, which contains 10bp of sequence 5’ of the *Otx2* “A” site, has little activity. Of the few GFP+ cells seen, less than half co-express Blimp1 (arrows). Asterisks mark Cherry+ cells that co-express Blimp1 (insets) and arrowheads mark GFP+/Blimp1- cells. **(D-D”)** The 139bp F3.1d element is active in explants and most GFP+ cells co-express Blimp1 (arrows, insets). GFP+ cells that do not co-express Blimp1 are marked with arrowheads (insets). **(E-E”)** The 97bp F3.1e construct, which is shorter on the 3’ end than F3.1d, has little activity. Arrowheads mark GFP+/Blimp1- cells (insets) and asterisks mark Cherry+/Blimp1+ cells (insets). Scale bars are 50 μm for panels and 10 μm for insets. **(F)** Plot of the average percentage of GFP+ and Cherry+ cells that co-express Blimp1. Error bars represent the S.D. Otx2 mut B, F3.1, F3.1a, F3.1c, and F3.1d show significant (*, unpaired t-test, P < 0.05) Blimp1 co-expression compared to cherry control. These five active elements do not show differences in Blimp1 co-expression (ANOVA, P > 0.05).

As *Otx2* is genetically upstream of *Blimp1*, we reasoned that the F3 element would contain one or more *Otx2* binding sites. We manually searched the 544bp F3 element sequence for consensus *Otx2* binding sites [37]. We identified two sites at 90bp (site A, AGATTA) and 256bp (site B, GGCTTA) from the 5’ end of F3 (Fig 4A and S1 Table). Read from the opposite DNA strand, these sites are similar to consensus binding sequences (T/CTAATCCC) for K50 type homeodomain proteins like Otx2 and Crx [38–42]. We designed constructs where 6bp of each potential *Otx2* binding site was mutated to be all A’s (S1 Table). These mutant *Otx2* constructs (F3 Otx mut A and B) were electroporated into retinal explants as above and screened after 1 DIV for GFP expression. We observed that F3 Otx mut A was unable to drive GFP expression (Fig 4A–4B”). Thus, the “A” Otx2 site is required for F3 enhancer activity. In contrast, the F3 Otx2 mut B construct drove robust GFP expression (data not shown). The GFP signal overlapped extensively with Blimp1 (83.7% ± 14.3% S.D.), which was significantly enriched compared to cherry control (unpaired t-test, P < 0.0001) (Fig 4F) and was similar to wild-type F3 co-expression values (Fig 2F). Therefore, the *Otx2* “B” site is not required for the activity of the F3 enhancer element. We then generated three derivatives of the F3 element (F3.1, F3.2, and F3.3) that each contained the “A” *Otx2* site and differing lengths of 5’ and 3’ flanking sequences (Fig 4A and S1 Table). The 228bp F3.1 construct contained both *Otx2* binding sites, with 35bp flanking the “A” site and 20bp flanking the “B” site. This construct was active in the retina (data not shown) and was significantly enriched (unpaired t-test, P < 0.05) for GFP+/Blimp1+ double labeled cells (77.4% ± 22.9% S.D.) compared to cherry control (Fig 4F). In contrast, the 125bp F3.2 construct that included only 27bp of sequence downstream of the *Otx2* “A” site showed no activity in retinal explants (data not shown and Fig 4F). Thus, sequences downstream of the *Otx2* “A” site are required for enhancer activity. We also built a 291bp hybrid F3.3 construct that included 12bp centered on the *Otx2* “A” site and the remaining sequence 3’ of the *Otx2* “B” site. The F3.3 construct showed no activity in retinal explants (data not shown and Fig 4F). Taken together, these data indicate that enhancer activity requires sequences downstream of the *Otx2* “A” site and upstream of the “B” site.

To further narrow the region, we cloned a series of five F3.1 derivatives that contained the “A” *Otx2* binding site and different lengths of 5’ and 3’ flanking sequence (Fig 4A and S1 Table). The 214bp F3.1a derivative lacked 14bp of sequence from the F3.1 parent construct at the 3’ end. Upon electroporation, we observed robust expression in the retina (data not shown). The GFP+ cells overlapped highly with Blimp1 (89.8% ± 12.1% S.D.), which was significantly enriched versus cherry control (unpaired t-test, P < 0.0001). The 203bp F3.1b construct contained only 10bp of sequence 5’ of the *Otx2* “A” site and was poorly expressed in retinal explants (Fig 4C–4C”). The number of GFP+ cells observed was low and they poorly overlapped with Blimp1 (41.5% ± 38% S.D.). This was not significantly different than cherry or TATA-only controls (Fig 4F). With the findings from above, these results indicated that sequence are required both 5’ and 3’ of the *Otx2* “A” site. We further truncated the 3’ end of the F3.1a construct to identify the sequences downstream of the *Otx2* “A” site required for enhancer activity. The 169bp F3.1c and 139bp F3.1d constructs both showed robust activity (Fig 4D–4D”) in the retina. They overlapped highly with Blimp1 (F3.1c- 75.3% ± 9.0% S.D, F3.1d- 74.4% ± 8.8% S.D) and both were significantly enriched over cherry control (unpaired t-tests, P < 0.001) (Fig 4F). In contrast, the 97bp F3.1e construct drove expression poorly with few GFP+ cells observed (Fig 4E–4E”). Those GFP+ cells present did not significantly co-express Blimp1 (23.5% ± 22.4% S.D.) compared to cherry controls (unpaired t-test, P > 0.05) (Fig 4E and 4F). Of the five constructs that showed significant enhancer activity, the percentage of GFP+ cells that co-expressed Blimp1 was not significantly different from each other (ANOVA, P > 0.05) (Fig 4F). From these experiments, the 139bp F3.1d element was the shortest sequence that recapitulated Blimp1 enhancer activity. These data indicated that the *Otx2* “A” site, upstream and downstream sequences are required for enhancer activity.

**Fig 5:**
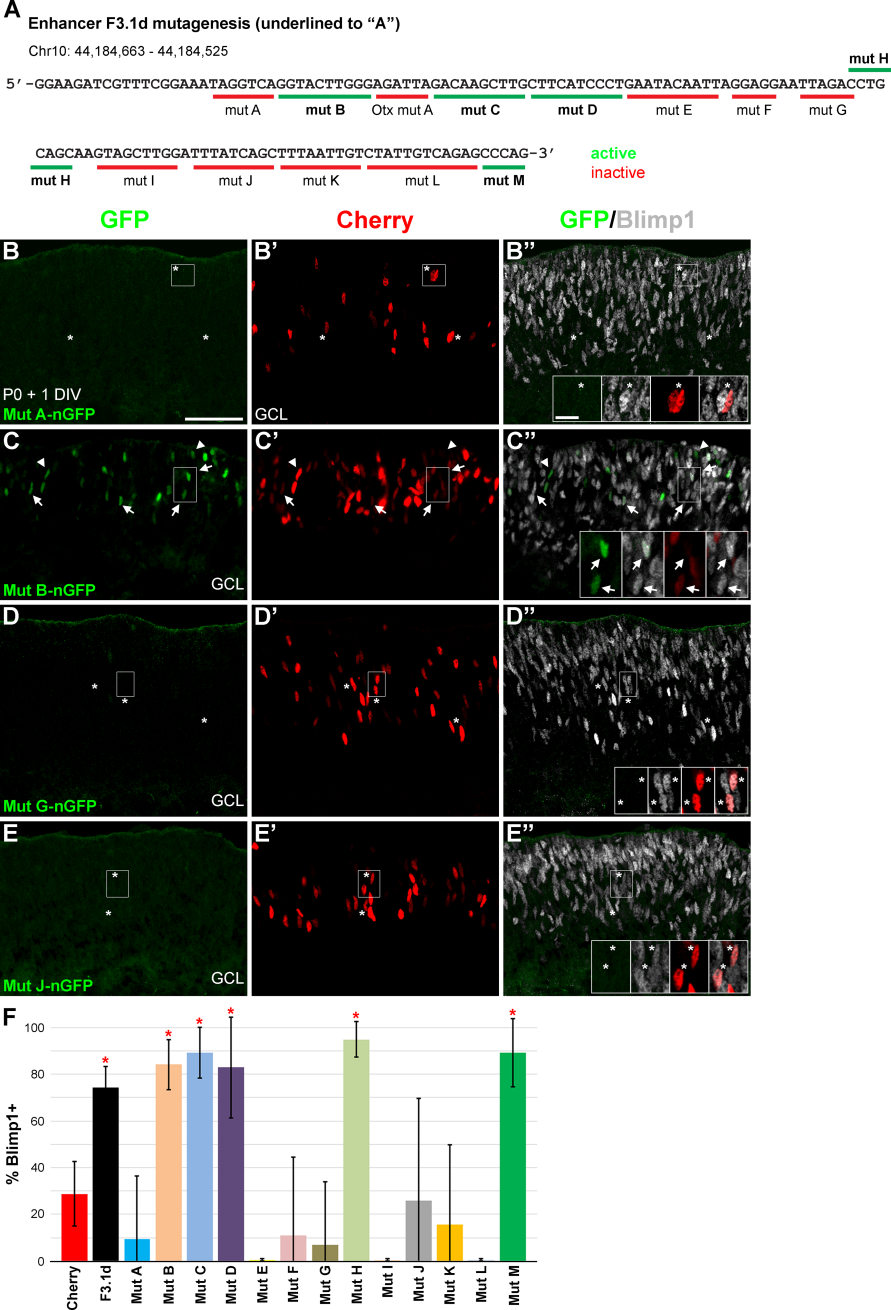
Tiled mutagenesis reveals four sequence regions necessary for enhancer activity. **(A)** The 139bp F3.1d sequence. Genomic coordinates are from the mouse mm9 assembly. The 14 regions subjected to mutagenesis (to A’s) are underlined and color-coded based on enhancer activity (green, active) (red, inactive) in explant assays cultured for 1 DIV. The Otx mut A result is from Fig 4. **(B-B”)** Mutation of the mut A sequence prevents F3.1d activity. Asterisks show Cherry+ cells (red) that co-express Blimp1 (grey, insets). **(C-C”)** In contrast, mutation of the adjacent mut B sequence does not block activity. Most of the GFP+ cells (green) co-express Blimp1 (arrows, insets). Arrowheads mark GFP+/Blimp1- cells. **(D-E”)** Mutating sequences at location G (D-D”) and J (E-E”) of the F3.1d element blocks activity. Asterisks show Cherry+ control cells that co-express Blimp1 (insets). Scale bar is 50 μm for panels and 10 μm for insets. **(F)** Plot of the average percentage of GFP+ and Cherry+ cells that co-express Blimp1. Error bars represent the S.D. Five of the mutations (B-D, H, M) do not affect activity, resulting in significantly more Blimp1 co-expression versus cherry control (*, unpaired t-test, P < 0.001). These active constructs have similar GFP and Blimp1 co-expression (ANOVA, P > 0.05). The remaining mutations (A, E-G, I-L) prevent activity, demonstrating that the sequences are required for enhancer activity.

Our enhancer deletion strategy suggested that at least three regions of the F3.1d element are required for its expression in explants. To identify these sequences, we systematically mutated the element to A’s in a tiled fashion (6-12bp at a time) to generate 13 new mutant constructs (Fig 5A and S1 Table). The expression of these constructs in retinal explants gave two patterns (Fig 5B–5E). First, there were five constructs (B, C, D, H, and M) that showed GFP expression. The GFP overlapped highly with Blimp1 in all of these cases (Fig 5C and 5F). These were significantly enriched compared to cherry control (unpaired t-tests, P < 0.001), but were not significantly different from one another (ANOVA, P > 0.05). The second pattern included eight constructs (A, E, F, G, I, J, K, and L) that either lacked GFP expression or showed a small number of weakly GFP+ cells that were not significantly enriched for Blimp1 co-expression compared to cherry controls (Fig 5B and 5D–5F). These data show that multiple regions of the F3.1d element are required for enhancer activity. This fits with the deletion analysis above, which implied that at least three discrete sites are needed for activity. Our tiled mutagenesis shows that there is a short essential region 5’ of the *Otx2* “A” site as predicted by the F3.1b construct data (Figs 4A and 5A). Downstream of this *Otx2* site are two stretches (23bp and 40bp) of required sequence separated by non-essential sequences (Fig 5A). The sequence at the 3’ end of the element was not required for activation. These data argue that there are at least four discrete sites in the F3.1d enhancer element that are each required for expression in the retina.

### Otx2 and other factors bind the F3.1d enhancer element

**Fig 6:**
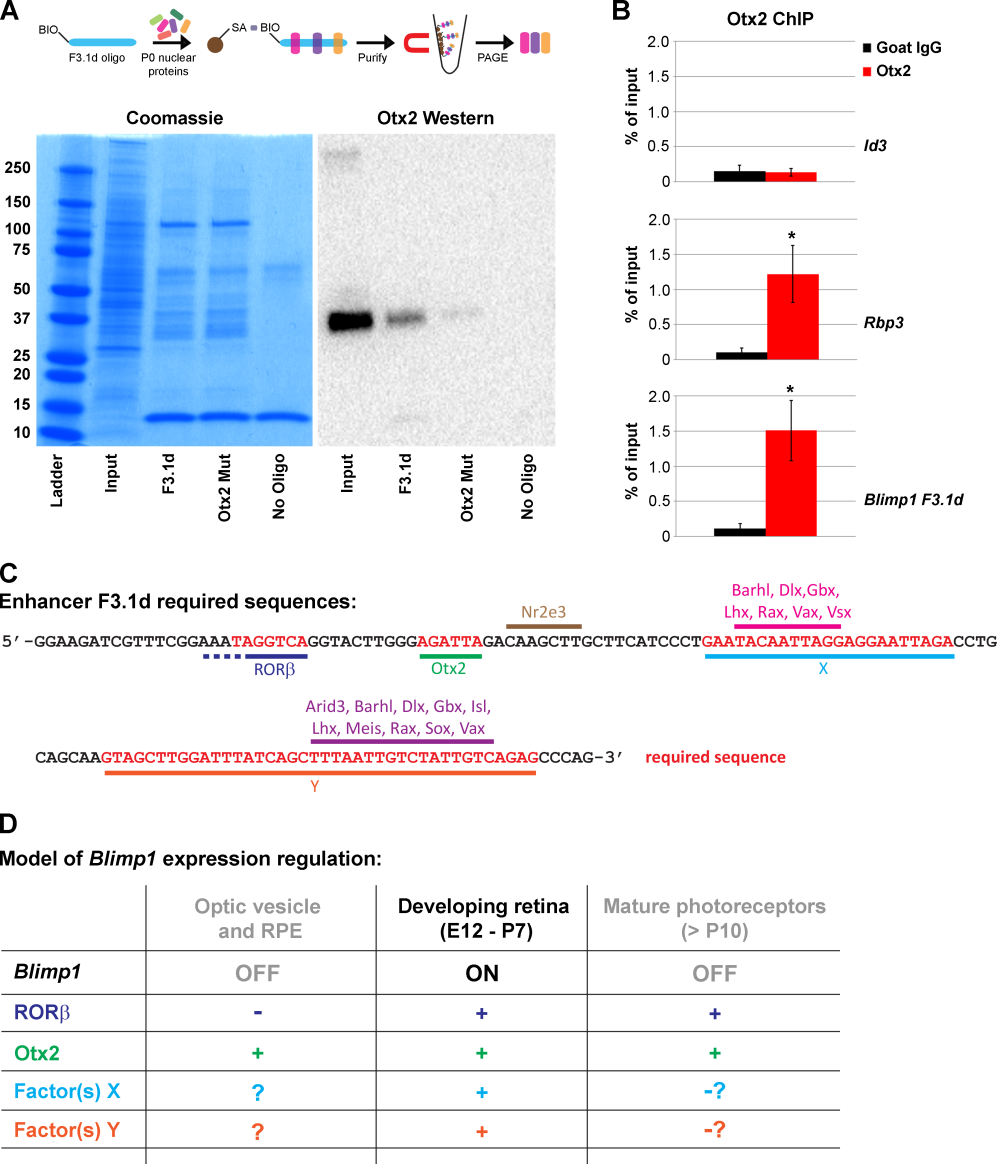
Otx2 and other transcription factors are required for *Blimp1* spatial and temporal enhancer activity. **(A)** Enhancer protein binding assays. 5’-biotinylated (BIO) oligonucleotides were made double stranded and bound to streptavidin (SA) coated dynabeads. P0 retinal nuclear protein lysate was incubated with the beads and the bound proteins purified and subjected to polyacrylamide gel electrophoresis (PAGE). (Left) A Coomassie gel containing unmodified nuclear lysate (input) and pulldowns with wild-type F3.1d oligos, *Otx2* binding site mutant oligos, and with unbound beads (no- oligo). The ladder band sizes are in kDa. Only a subset of the input proteins bind to the F3.1d and Otx2 mutant oligos. The no-oligo condition showed modest non-specific bead binding background. The intense band at 12 kDa represents the SA boiled from the dynabeads. (Right) Western blot of these samples shows a robust Otx2 signal in the input lane at 32–35 kDa (expected size of 31-32kDa). The F3.1d wild-type sequence pulls down Otx2 more strongly than the mutant oligo. Otx2 is not pulled down in the no-oligo condition. This shows that Otx2 is preferentially binding to its consensus site and not elsewhere in the sequence. **(B)** Otx2 chromatin immunoprecipitation (ChIP) on P0 retinal cells as a percentage of the input signal. Error bars represent S. D. The *Id3* promoter region lacks *Otx2* binding sites and Otx2 (red) does not pulldown the *Id3* promoter more than goat IgG control (black). *Rbp3* is a known Otx2 target and is immunoprecipitated strongly by Otx2 versus goat IgG (*, unpaired t-test, P < 0.03). Otx2 also pulls down the *Blimp1* F3.1d enhancer region significantly (*, unpaired t-test, P < 0.02) compared to IgG control. **(C)** F3.1d sequence showing required (red) sequence regions. JASPAR was used to predict transcription factor binding (underlined). Factors with a 90% relative score threshold that are expressed in the P2 retina are shown. The 5’ most required sequence strongly matches a ROR response element. Rod-specific Nr2e3 is predicted to bind in non-essential sequence, while several homeodomain transcription factors are predicted to bind in subsections of the X and Y regions. Additional candidate transcription factors are seen when the score threshold is lowered to 80% (S2 Table). **(D)** Multi-component regulation of *Blimp1* gene expression. Other work has implicated RORβ as the ROR family member bound to F3.1d [43]. Otx2, RORβ, and factors bound to the X and Y sites are required for expression. At early stages of eye development and within the RPE, Otx2 is present while RORβ is absent, preventing *Blimp1* expression. Later, Otx2, RORβ, and activators that bind X and Y must be present to achieve robust expression in photoreceptors. In contrast to *Blimp1* and its enhancer, Otx2 and RORβ remain expressed in mature photoreceptors. This suggests that temporal regulation of the enhancer occurs through the X and Y sites. This could be due to the loss of activating factors and/or the accumulation of silencing factors that are able to supersede or compete with the activators.

The tiled mutagenesis experiments suggested that four regions of the F3.1d enhancer are required for activity. Since *Blimp1* is genetically downstream of *Otx2* in the retina, we first tested whether Otx2 could bind the F3.1d element. To do this, we generated double stranded oligonucleotides to the F3.1d element and biotinylated the 5’ end of one strand. Similar oligos were made with the *Otx2* binding site mutated to be all A’s (S1 Table). These were bound to streptavidin-coated dynabeads and incubated with newborn retinal nuclear lysate (Fig 6A). After purification, the protein bound to the oligos was subjected to polyacrylamide gel electrophoresis and used for Coomassie stains or Western blot. As a negative control, we used beads that were not incubated with biotinylated oligos. Few proteins stuck non-specifically to the dynabeads (Fig 6A). The pulldowns with wild-type F3.1d and *Otx2* mutant oligos showed that only a subset of retinal nuclear proteins can bind these DNA sequences (Fig 6A). It was quite difficult to appreciate banding differences between the control and mutant oligo pulldowns by Coomassie staining. However, upon Western blotting with anti-Otx2 antibodies, the differences were apparent (Fig 6A). As expected, there was strong Otx2 signal in the input lane and no signal in the negative control (no-oligo) condition. The Otx2 signal was much stronger with the F3.1d wild-type pulldowns compared to the *Otx2* mutant oligos (Fig 6A). This suggests that Otx2 is binding at the predicted site. To test whether Otx2 binds elsewhere in the F3.1d region, we synthesized shorter (60bp) biotinylated oligos encompassing the sequences immediately downstream of the *Otx2* binding site (S1 Table). Western blot did not reveal Otx2 signal in these pulldowns (data not shown), arguing that Otx2 does not bind elsewhere in the F3.1d sequence.

We then conducted chromatin immunoprecipitation (ChIP) experiments on newborn retinal tissue (Fig 6B) to test whether Otx2 binds the *Blimp1* enhancer *in vivo*. We first examined the *Id3* gene promoter region that lacks *Otx2* binding sites [23, 37] as a negative control. As expected, we saw no differences when we immunoprecipitated with goat anti-Otx2 antibodies versus pan-specific goat IgG molecules (Fig 6B). As a positive control, we examined the *Rbp3* gene, a known Otx2 target [23, 44]. We saw strong Otx2 enrichment compared to goat IgG control (unpaired t-test, P < 0.03). With primers to the F3.1d enhancer region of the genome, we saw strong Otx2 enrichment compared to goat IgG (unpaired t-test, P < 0.02) (Fig 6B). These data argue that Otx2 binds the F3.1d enhancer *in vivo*.

Next, we used JASPAR (http://jaspar.genereg.net/) [27, 28] to search the well conserved F3.1d sequence for potential transcription factor binding sites (Fig 6C and S2 Fig). We used the core vertebrata matrix and set the relative profile score threshold to 90% (S2 Table). Using previously generated RNA-seq data from the P2 retina [29], we excluded those genes that showed no expression in the retina. JASPAR predicted the *Otx2* binding site and the sequence upstream matched an ROR response element (AGGTCA with 5bp of A/T rich upstream sequence) [45] (Fig 6C). All three ROR genes (*Rora*, *Rorb*, and *Rorc*) are made in the P2 retina, but *Rorb* is expressed at the highest levels [29]. Thus, we attempted ChIP with multiple RORβ antibodies, but none of them gave reproducible results (data not shown). Most of the JASPAR transcription factor predictions clustered in the two longer required sequence regions (X and Y), with one exception (Fig 6C and S2 Table). There was an *Nr2e3* binding site in a stretch of sequence that was not required for expression of the F3.1d element (Fig 6C). The 5’ portion of region X had strong predictions for Q50 type homeodomain transcription factor binding [38, 46], but lacked predictions for the 3’ end (Fig 6C). However, when we lowered the score threshold to 80%, the entire F3.1d element was saturated with potential binders (S2 Table). This lowered stringency predicted additional Q50 homeodomain binding sites along with *Arid3*, *Mzf*, *Otx2*, *Tcf3*, and *Tead* sites in region X (S2 Table). The longer Y region had strong predictions near the 3’ end, but lacked them for the 5’ portion of the region (Fig 6C and S2 Table). Like the X region, this included predictions for Q50 homeodomain factors. We also saw strong predictions for *Meis* and *Sox* genes. Lowering the score threshold increased the predictions for the 5’ end of the Y region and indicated that *Cux*, *Hmx*, *Otx2*, *Nfic*, *Nfix*, *Nr2e3*, *Nrl*, and *Tbx2* transcription factors may bind within the region (S2 Table). As described above, it is unlikely that Otx2 is regulating expression through binding the X or Y regions. Our mutagenesis data along with the JASPAR predictions argue that three or more different transcription factors are binding to at least four discrete sites to activate expression of the F3.1d enhancer element.

## Discussion

We identified several candidate retina-specific *Blimp1* enhancers with DHS sequencing. Using relatively high-throughput explant culture assays, we identified a single non-coding region that recapitulated *Blimp1* expression both in explants and *in vivo*. Closer scrutiny revealed that this enhancer is regulated by Otx2, a ROR factor, and unknown factors that bind at two additional discrete regions within this sequence. This argues that the combinatorial action of at least three transcription factors is required for *Blimp1* enhancer activity. This multifactorial gene regulatory network can account for how Blimp1 expression differs from Otx2 and leads to multiple fate outcomes from this population. Further dissection of the gene regulatory network that controls *Blimp1* expression is needed to understand the mechanisms that set the balance of cell fates during development.

### Identification of a *Blimp1* enhancer

We took advantage of prior profiling of DHS sites from whole retinas at different developmental stages [25]. This was particularly useful for identifying potential *Blimp1* enhancers, as this gene is expressed by a large fraction of retinal cells during development. Of the two candidate DHS peaks that showed expression in the retina (F and I), only the F sequence showed strong co-expression with Blimp1 protein in our assays. The poorly conserved DHS region I was located in an intron of the *Atg5* gene and showed essentially random expression in explants. This DHS may contain generic activating sequences or contribute to *Atg5* regulation. The remaining DHS regions did not display enhancer activity and their function remains unknown. DHS A is near an alternative transcription start site for *Blimp1* [47] that is not used in the retina (data not shown) and may therefore mark an extended promoter region instead of an enhancer.

DHS F and its smaller derivatives showed about 80–85% overlap with Blimp1 protein in our assays. This incomplete overlap is most likely the result of transient *Blimp1* expression in non-photoreceptor cell types. In embryonic F3-nGFP transgenic mice, GFP+ cells that did not make Blimp1 protein were not randomly distributed, rather they co-localized with the amacrine/horizontal precursor marker Ptf1a. At P7, a small fraction of GFP+ cells co-expressed bipolar and to a lesser extent amacrine markers. We did not observe GFP in ganglion cells or Müller glia. These observations match our prior lineage tracing data, which showed that *Blimp1* is transiently expressed by cells that contribute to amacrine, horizontal, and bipolar cell identities [23]. When accounting for the slow turnover of GFP protein, our data argue that the GFP pattern is a highly accurate representation of *Blimp1* expression history in the retina. Since GFP expression was not seen in other Blimp1+ cell populations, such as developing vascular endothelial cells, the DHS F region likely functions as a retina-specific *Blimp1* enhancer.

Blimp1 is made between about E12.5 and P10 in the retina. Consistent with accurate temporal control, we first observed GFP at about E12.5 in transgenic mice. Newborn electroporated explants grown for 10 days in culture essentially lacked GFP and transgenic mice showed the expected progressive loss of GFP expression after P7. However, we observed some weakly GFP+ cells in the adult retina of transgenic mice. There are several explanations for this modest persistent GFP signal. First, *Blimp1* may be expressed below our ability to reliably detect in the adult wild-type retina, making GFP the more sensitive and accurate read-out. Second, persistent weak GFP expression could be an artifact of the transgene integration site or the number of copies present. In support of this possibility, weak GFP expression in adult transgenic mice was highly mosaic. Third, it is possible that the full silencing of the transgene requires cis-regulatory sequences outside of the F3 region. Lastly, miRNA targeting of *Blimp1* transcripts may be required for complete silencing. While miRNA can influence *Blimp1* expression [43, 48–56], the paucity of GFP expression in long-term explant cultures and adult transgenic mice suggests that *Blimp1* expression in the retina is primarily regulated at the level of transcription. Despite modest GFP expression seen in adult transgenic animals, the *Blimp1* enhancer demonstrates a high degree of temporal specificity.

Wang and colleagues [43] used a different approach to identify *Blimp1* enhancers and found a region that is a subset of the F3.1d sequence we describe. Similar to our findings, their 108bp enhancer region (detailed further below) had about 90% overlap with Blimp1 protein in electroporated retinas. Using Cre-Lox based lineage tracing in postnatal electroporated eyes, their *Blimp1* enhancer marked photoreceptors, amacrine cells, and some bipolar cells. They similarly observed weakly GFP+ cells in older animals following electroporation, arguing against transgene integration artifacts as a cause of persistent GFP expression in our experiments. Wang et al [43] also conducted CRISPR/Cas9 *in vivo* electroporation experiments to remove the endogenous *Blimp1* enhancer from the postnatal retina. They observed a phenotype similar to that seen in *Blimp1* mutants, arguing that the enhancer region is necessary for *Blimp1* expression in the retina.

### ROR and Otx2 regulation of the *Blimp1* enhancer and photoreceptor development

The *Blimp1* enhancer sequence contains highly conserved ROR and Otx2 binding sites that are necessary for enhancer activity. Using ChIP, we showed that Otx2 can bind the enhancer *in vivo*. Wang and colleagues [43] showed that knocking down either *Rorb* or *Otx2* strongly reduced enhancer activity. These findings suggest that both Otx2 and RORβ are necessary for *Blimp1* expression during retinal development (Fig 6D). Early in retinal development, Otx2 is present in the optic vesicle, the nascent optic cup, and the RPE [14–16]. RORβ is not expressed at these early stages or by the RPE [57], and Otx2 is not sufficient to activate *Blimp1* at this time (Fig 6D). After E12.5, RORβ becomes widely expressed in progenitors [57–61] while Otx2 becomes activated by progenitors in their last cell cycle that can adopt photoreceptor, bipolar, horizontal, and amacrine fates. Thus, the overlap of RORβ and Otx2 allows the *Blimp1* enhancer to be activated at the appropriate time and place (Fig 6D). *Blimp1* and its enhancer show little if any expression in mature photoreceptors, but Otx2 and RORβ expression is maintained in these cells [60, 62, 63] (Fig 6D). This argues that Otx2 and RORβ are necessary, but not sufficient for *Blimp1* regulation.

Consistent with ROR and Otx2 being only part of the *Blimp1* regulatory network, our tiled mutagenesis experiments revealed that two other sequence blocks (X and Y) are required for *Blimp1* enhancer activity (Fig 6C). Here, our data diverges from that of Wang and colleagues [43], painting a more complex regulatory picture. Their 108bp minimum element begins just 5’ of the ROR element and ends in the middle of the Y sequence, such that 13bp of the Y region is absent (S2 Fig). This contrasts with our results, where we showed the sequence immediately 3’ of their element (Fig 5 and S2 Fig) is required for activity. Wang *et al* [43] found that mutagenesis of selected bases, particularly in the Y area, had only a modest effect on enhancer activity. In contrast, we observed that multiple mutations in the X and Y regions fully prevented enhancer activity. The reason for these differences is unclear, but we made more drastic sequence changes (strings of A’s), which could more strongly affect activity. The X and Y regions are each rather broad, suggesting that they contain multiple required binding sites. For example, mutations I and L prevented enhancer activity (Fig 5 and S2 Fig), but it is unlikely that this represents one overlapping binding site as there are 19bp separating these mutations. There are no predicted ROR binding sites in the X or Y regions and Otx2 does not specifically bind these regions in our pulldown assays. Thus, we can expand our simplified model to have additional activating components that bind regions X and Y (Fig 6D). The identity of the factors that bind regions X and Y is currently unknown (see below). At early stages or in the RPE, enhancer activity will be blocked regardless of whether factors X and Y are present (Fig 6D). During photoreceptor genesis, factors X and Y are needed for *Blimp1* enhancer activity (Fig 6D). It is possible that Otx2 and RORβ are sufficient to activate *Blimp1* below the level of protein detection, which would allow highly sensitive recombination systems or long-lived reporter proteins to mark a broader expression pattern. The *Blimp1* lineage pattern closely matches what is predicted from the intersection of Otx2 and RORβ; nonetheless, our data argue that binding of factors X and Y are needed for full enhancer activity and detectable Blimp1 protein expression. To downregulate the enhancer in adult photoreceptors, the X or Y factors could be lost (Fig 6D) or outcompeted by negative factor binding.

Wang and colleagues [43] work suggests that the *Blimp1* enhancer acts non-redundantly to activate *Blimp1* transcription. This implies that Otx2 and RORβ are essential regulators of photoreceptor fate. This is certainly true for *Otx2*, as mutants lack photoreceptors and other cell types in the retina [18–20]. In contrast, *Rorb* is expressed broadly and mutant mice have a complex phenotype, primarily involving photoreceptors and amacrine cells [59–61, 63, 64]. These mutants do not show major changes in *Otx2* expression or a fate shift of photoreceptors to bipolar cells. This argues that *Blimp1* transcription is still present in these mutants, although this has not been tested directly. Since the ROR binding site is critical for *Blimp1* enhancer activity, other ROR genes (*Rora* and *Rorc*) may compensate or act redundantly with RORβ to regulate the *Blimp1* enhancer. Although RORβ can act with Otx2 to induce rod-specific *Nrl* expression [65, 66], it can also combine with Foxn4 to activate the amacrine and horizontal precursor-specific regulator *Ptf1a* [60]. These findings and *Blimp1* lineage tracing results (see above) argue that the intersection of Otx2 and RORβ in undifferentiated cells does not convey photoreceptor identity nor *Blimp1* spatial and temporal specificity. Instead, these factors function permissively to allow photoreceptor genes like *Blimp1* and *Nrl* to become expressed. This is consistent with our *Blimp1* enhancer data and shows that additional factors are needed to fully activate and stabilize photoreceptor gene regulatory networks at early developmental stages.

### Other factors that regulate the *Blimp1* enhancer

Otx2 and ROR transcription factors can combine to impart competence to activate photoreceptor gene regulatory networks. The *Blimp1* enhancer requires these factors and an unknown number of additional regulators that bind the X and Y sequences for its activation. We used JASPAR to predict what transcription factors may be binding the X and Y sites (Fig 6C). This mostly predicted Q50 type homeodomain transcription factors. The best candidate predicted to bind both X and Y sites was *Rax*, a Q50 homeodomain transcription factor expressed in progenitors, glia, and photoreceptors [17, 67–76]. Similar to Blimp1, the levels of *Rax* decline as photoreceptors mature. Loss of *Rax* during mouse neurogenesis inhibits *Otx2* expression while late loss affects cone survival [17, 70]. Thus, it is unclear whether *Rax* activates *Blimp1* downstream of its role in controlling *Otx2* expression. The strong downregulation of *Rax* in mature photoreceptors could explain why *Blimp1* enhancer activity declines with age. The Q50 LIM homeodomain factor *Lhx2* may activate the *Blimp1* enhancer. When *Lhx2* is deleted at the onset of neurogenesis (E11.5), there is a reduction in *Blimp1* expression [77]. However, these mutants also have a strong reduction in *Rorb* expression. Deletion of *Lhx2* at E15.5 results in excess photoreceptors and no evidence of a fate switch to bipolar cells, suggesting that *Blimp1* is still transcribed [78]. Thus, whether Lhx2 directly activates the *Blimp1* enhancer remains unclear. Other Q50 homeodomain factors, like *Vsx2*, are expressed by non-photoreceptor cell types in the retina and seem likely to repress the *Blimp1* enhancer. None of the mutations in the X or Y region resulted in strong non-specific expression, arguing that silencers compete with activators that bind the same regions. The interplay of activators and repressors at the *Blimp1* enhancer may explain why the DHS F peak is still present in the adult retina despite the lack of *Blimp1* transcription. Additional work is needed to characterize if any of these candidate factors bind the F3.1d element and whether perturbing their function in the retina affects *Blimp1* expression and the proportion of Otx2+ cells that adopt photoreceptor identity.

## Conclusions

The relative numbers and cell types formed changes considerably throughout retinal development. How this stochastic fate choice process is regulated remains largely unknown. Blimp1 inhibits bipolar cell development in a subset of cells that can adopt photoreceptor and bipolar cell fates. To understand how this fate choice balance is achieved, we investigated the gene regulatory network that controls *Blimp1*. We used DHS profiling to identify an enhancer element that recapitulates *Blimp1* expression the retina. Closer analysis revealed four discrete sequences in the *Blimp1* enhancer are required for its activity, suggesting the gene regulatory network that controls this enhancer is multifactorial and changes over time. Our data show that Otx2 and ROR transcription factors are necessary, but not sufficient to regulate *Blimp1*. The requirement of additional factors explains how *Blimp1* expression diverges from Otx2 and RORβ during development to facilitate cell type diversification. Even modest fluctuations in the levels or timing of these additional factors could affect how many cells activate *Blimp1* and the intensity and duration of gene expression. This would affect the probability of forming photoreceptors and bipolar cells, partially explaining the stochastic nature of mammalian retinal development.

## Supporting information

S1 Fig DNase hypersensitivity site (DHS) sequencing data at the mouse *Blimp1* locus. **(A)** UCSC Genome Browser with ENCODE tracks (mm9 assembly) showing DHS seq data from P0, P7, and P56 retina over a 235kb region. We identified 9 DHS peaks (A-I, shaded red or green), some of which showed differential signal based on age (*e*.*g*. C). DHS A was near an alternative *Blimp1* promoter while DHS I was in the intron of the *Atg5* gene. We excluded the peak at the *Blimp1* transcription start site. The sites showed high evolutionary conservation except for A and I. **(B-C)** Zoomed in views of the boxed (B, blue) (C, orange) regions showing the candidate sites in more detail. **(D)** ENCODE DHS data from the retina and other tissues. *Blimp1* is expressed by P0 and P7 retina and in activated T-regulatory cells, but is absent from the cerebellum, brain and the adult retina. The brain DHS sites do not overlap with the retinal ones, except for site A. In activated T-regulatory cells (blue), the DHS peaks A and G are shared with the retina. There are separate DHS peaks that may uniquely drive *Blimp1* expression in T cells.
(TIF)

S2 Fig Sequence conservation of the Blimp1 F3.1d enhancer element. From the UCSC Genome Browser. Genomic coordinates are from the mouse mm9 assembly. **(A)** Shown at the top is vertebrate conservation, with upward peaks indicating high conservation. Plotted vertically is the sequence of the same region in 50 vertebrate species. The ROR, Otx2, X and Y regions are shaded. Also indicated are the 14 mutations made in the F3.1d sequence (green mutants show enhancer activity while red ones prevent activity) and the 108bp sequence (black line) from Wang and colleagues [43] that mimics *Blimp1* expression. The Otx2 and ROR areas are very highly conserved in all species that align. The X region is highly conserved on the 3’ side and is divided by a 26bp gap in most species. This X region may be two distinct areas in other species. The Y region is especially conserved in the middle, but is generally well conserved throughout. **(B)** UCSC Genome Browser with the HOMER http://homer.ucsd.edu/homer/ analysis track showing potential transcription factor binding sites throughout the F3.1d sequence. The X and Y regions are predicted to bind homeodomain and Sox transcription factors, similar to what is seen by JASPAR analysis (Fig 6C and S2 Table).
(TIF)

S1 Table DNA sequences used. A list of all oligonucleotide and enhancer sequences with genomic coordinates (mouse mm9 assembly) where applicable.
(XLSX)

S2 Table JASPAR analysis of the F3.1d enhancer element. Predictions are grouped based on the relative score threshold (90% left, 80% right).
(XLSX)
