# Supplementary figures and images for: Combinatorial regulation of a Blimp1 (Prdm1) enhancer in the mouse retina
Source: PLoS One. 2017 Aug 22;12(8):e0176905. doi: 10.1371/journal.pone.0176905 (PMC5568747; doi:10.1371/journal.pone.0176905)

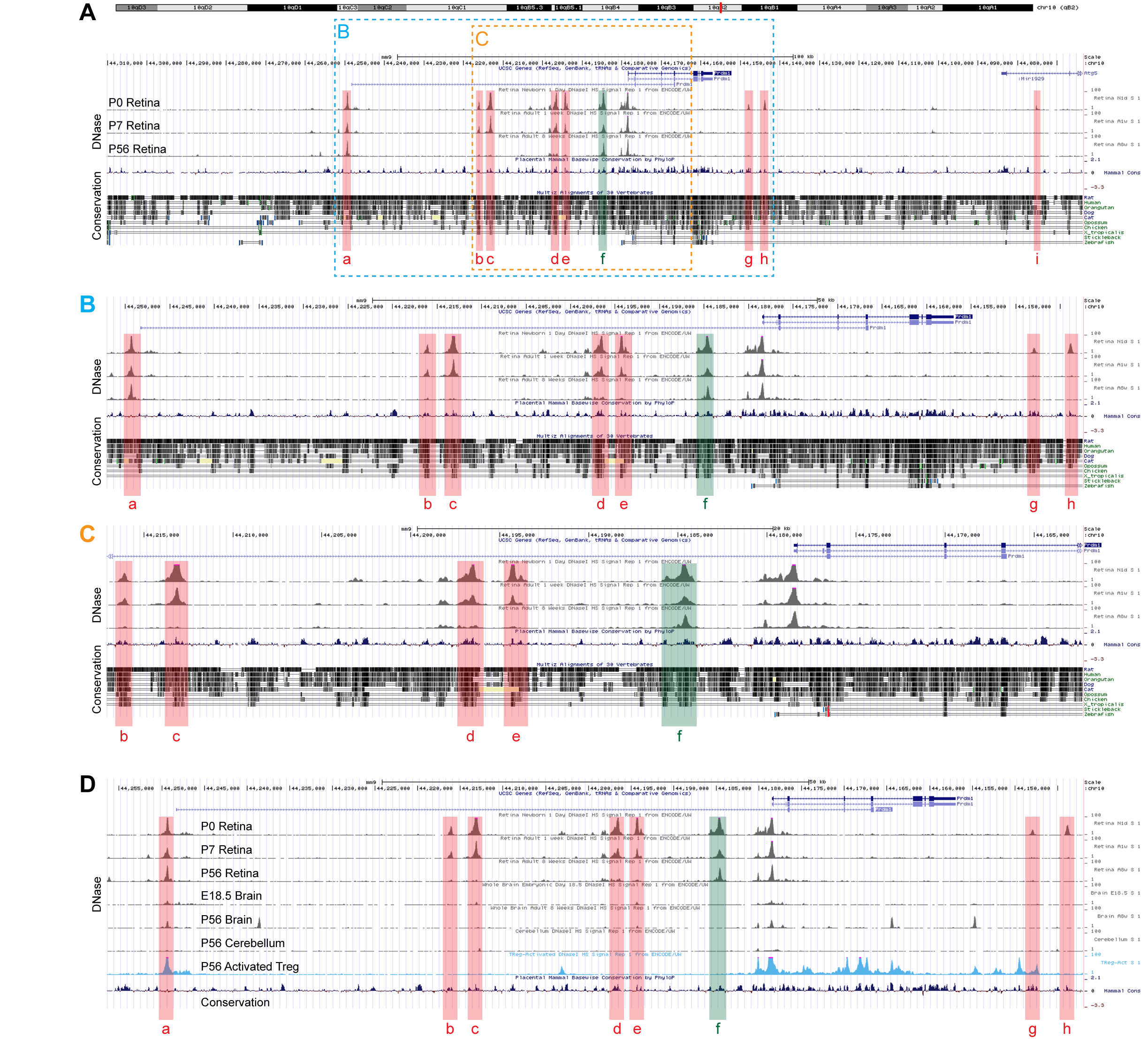

Supplement: S1 Fig — (A) UCSC Genome Browser with ENCODE tracks (mm9 assembly) showing DHS seq data from P0, P7, and P56 retina over a 235kb region. We identified 9 DHS peaks (A-I, shaded red or green), some of which showed differential signal based on age (e.g. C). DHS A was near an alternative Blimp1 promoter while DHS I was in the intron of the Atg5 gene. We excluded the peak at the Blimp1 transcription start site. The sites showed high evolutionary conservation except for A and I. (B-C) Zoomed in views of the boxed (B, blue) (C, orange) regions showing the candidate sites in more detail. (D) ENCODE DHS data from the retina and other tissues. Blimp1 is expressed by P0 and P7 retina and in activated T-regulatory cells, but is absent from the cerebellum, brain and the adult retina. The brain DHS sites do not overlap with the retinal ones, except for site A. In activated T-regulatory cells (blue), the DHS peaks A and G are shared with the retina. There are separate DHS peaks that may uniquely drive Blimp1 expression in T cells. (TIF) [file pone.0176905.s001.tif]

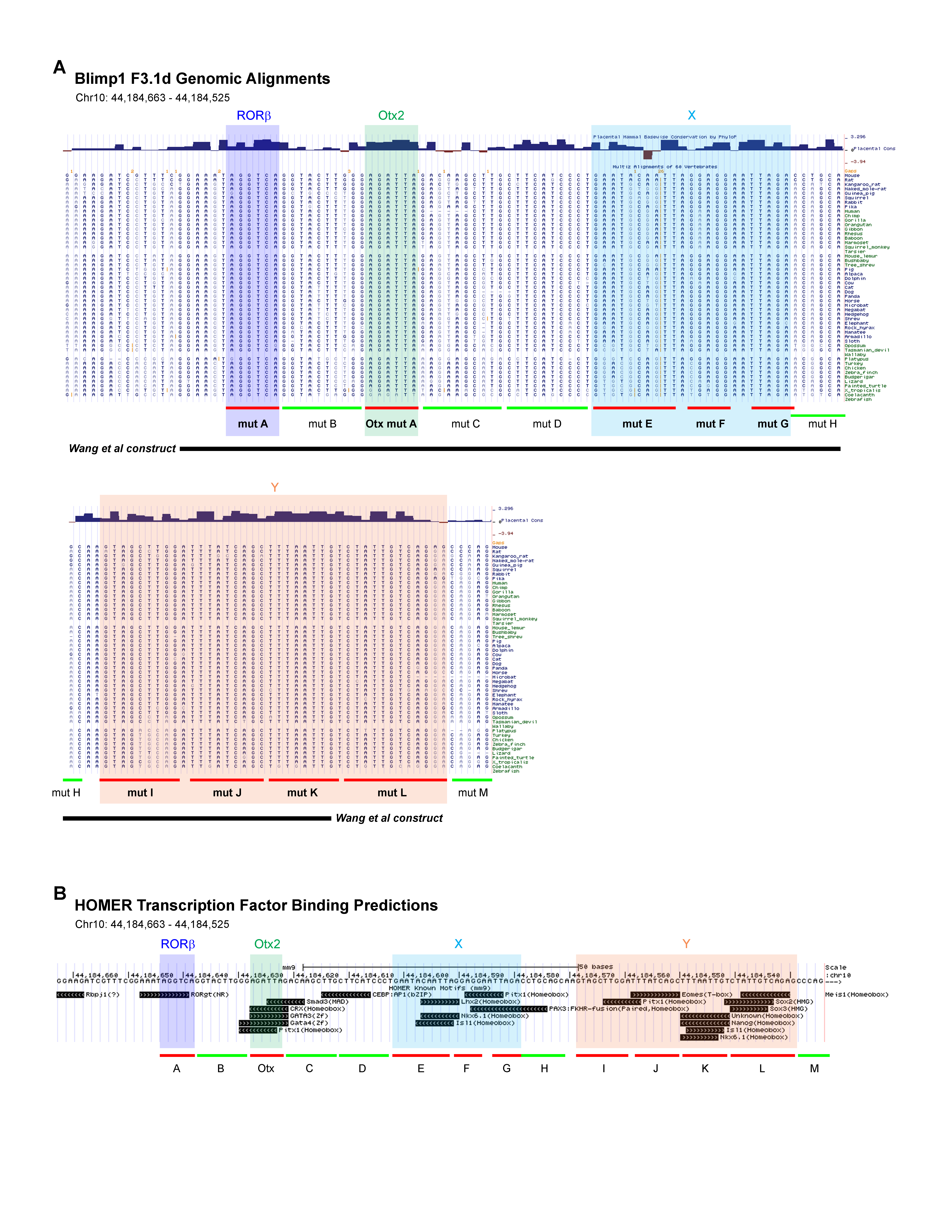

Supplement: S2 Fig — From the UCSC Genome Browser. Genomic coordinates are from the mouse mm9 assembly. (A) Shown at the top is vertebrate conservation, with upward peaks indicating high conservation. Plotted vertically is the sequence of the same region in 50 vertebrate species. The ROR, Otx2, X and Y regions are shaded. Also indicated are the 14 mutations made in the F3.1d sequence (green mutants show enhancer activity while red ones prevent activity) and the 108bp sequence (black line) from Wang and colleagues [43] that mimics Blimp1 expression. The Otx2 and ROR areas are very highly conserved in all species that align. The X region is highly conserved on the 3’ side and is divided by a 26bp gap in most species. This X region may be two distinct areas in other species. The Y region is especially conserved in the middle, but is generally well conserved throughout. (B) UCSC Genome Browser with the HOMER http://homer.ucsd.edu/homer/ analysis track showing potential transcription factor binding sites throughout the F3.1d sequence. The X and Y regions are predicted to bind homeodomain and Sox transcription factors, similar to what is seen by JASPAR analysis (Fig 6C and S2 Table). (TIF) [file pone.0176905.s002.tif]
